# Strongyloidiasis Infection As the Cause of Pancreatitis

**DOI:** 10.7759/cureus.24097

**Published:** 2022-04-13

**Authors:** Jorge Verdecia, Andres Martinez, Malleswari Ravi

**Affiliations:** 1 Infectious Diseases, University of Florida College of Medicine, Jacksonville, USA; 2 Internal Medicine, University of Florida College of Medicine, Jacksonville, USA

**Keywords:** peripheral eosinophilia, whipple's procedure, ivermectin, pancreatitis, strongyloides

## Abstract

Strongyloidiasis is endemic to socioeconomically disadvantaged tropics and subtropics, primarily as an asymptomatic carriage. It is seen in developed nations among the underprivileged. We describe a case of an Asian immigrant with eosinophilia, common bile duct stricture, cholangitis, and pancreatitis with *Strongyloides *spp*.* found on duodenal biopsy in the setting of steroid exposure.

## Introduction

*Strongyloides stercoralis (S. stercoralis) *is an intestinal nematode that affects 100 million people worldwide [1]. Intestinal nematodes have a limited life span inside the host, and their life cycle requires development in soil [1]. *S. stercoralis* is unique from other soil-transmitted helminths (STHs) with a complex life cycle, free-living and parasitic phases [1,2]. 

Most primary infections occur when filariform larvae in the contaminated soil penetrate the human host skin. Rarely do infections occur via the fecal-oral route. The larvae from the skin enter the venous circulation into the heart and lungs, then migrate through the alveoli [2]. Throughout this process, it continues to mature and eventually gets coughed up and swallowed into the small intestine, where mature female worms lay eggs. Rhabditiform larvae that hatch from the eggs are either excreted in the stool or remain in the intestine, developing into filariform larvae. The intestinal filariform larvae can burrow through the colonic walls or perianal skin directly into the duodenum or enter the blood, causing autoinfection [1].

The medical importance of this auto-infective cycle is that the parasite can perpetuate its life cycle independent of the external environment, and a host can remain infected lifelong [1]. Additionally, imbalance in the host immune system, as occurs with corticosteroids or HTLV-1, can result in accelerated autoinfection with large amounts of parasites leading to hyper infection syndrome [2]. This overwhelming parasitic burden can cause the filariform larva to lodge in organs and structures atypical for* S. stercoralis* [1]. Further complications include translocation of bacteria due to migration of larvae from the intestine, causing recurrent gram-negative bacteremia or sepsis, including meningitis. Disseminated infection with larvae invading numerous organs has a mortality rate that can approach 100% [1].

## Case presentation

The patient is a 63-year-old male who migrated from Cambodia 25 years ago. He has a past medical history of gastroesophageal reflux disease (GERD). He presented to his primary care physician with complaints of bloating, reflux, epigastric abdominal pain, and discoloration of urine. Patient-reported that the symptoms were worsening within the past year. Of note, he had bronchitis approximately three months before this presentation, for which he received a course of steroids and antibiotics. After admission, the patient developed fevers, and the workup showed multiple lab abnormalities (Table 1). Abdominal ultrasound showed heterogeneity of the visualized head of the pancreas with a prominence of the pancreatic duct, dilated common bile duct (CBD), moderately dilated intrahepatic ductal structures, and diffusely enlarged gallbladder with a large amount of sludge and small stones. He was referred to a gastroenterologist, but the patient presented to the emergency room for severe abdominal pain, anorexia, and weight loss before his appointment. The differential diagnosis for the stricture at the common bile duct included pancreatic malignancy versus inflammation at the duodenal ampulla and pancreatic duct from unknown causes. 

**Table 1:** Pertinent laboratories before and on admission ALT: alanine transaminase, AST: aspartate aminotransferase, ALP: alkaline phosphatase, T-bili: total bilirubin

Labs	Prior to admission	On admission
White Blood cells	14380/mm^3^	13550/mm^3^
ALT	47 IU/L	140 IU/L
AST	33 IU/L	88 IU/L
ALP	170 IU/L	439 IU/L
T – Bili	5 mg/dL	1.1 mg/dL
Lipase	130 U/L (7-60)	N/A
CA 19-9	64 U/mL (0-35)	N/A

**Figure 1:**
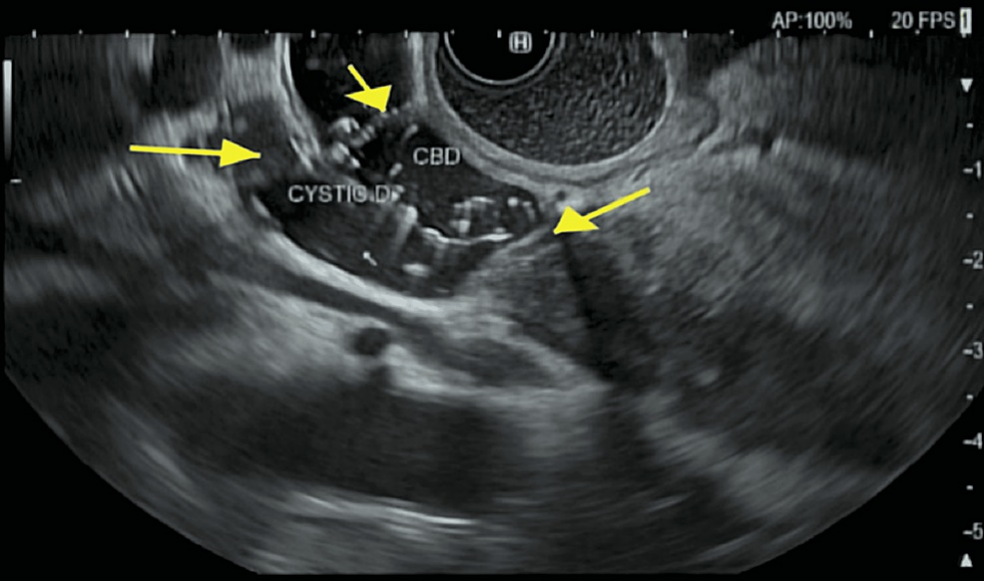
There was dilation in the common bile duct and the cystic duct, which measured 12 mm. The cut-off is right at the beginning of its intrapancreatic portion. Extensive hyperechoic material consistent with sludge was visualized endosonographically in the common bile duct, the cystic duct, and the gallbladder. The peri-ampullary portion of the biliary duct and the pancreatic duct was intact, without dilation.

**Figure 2:**
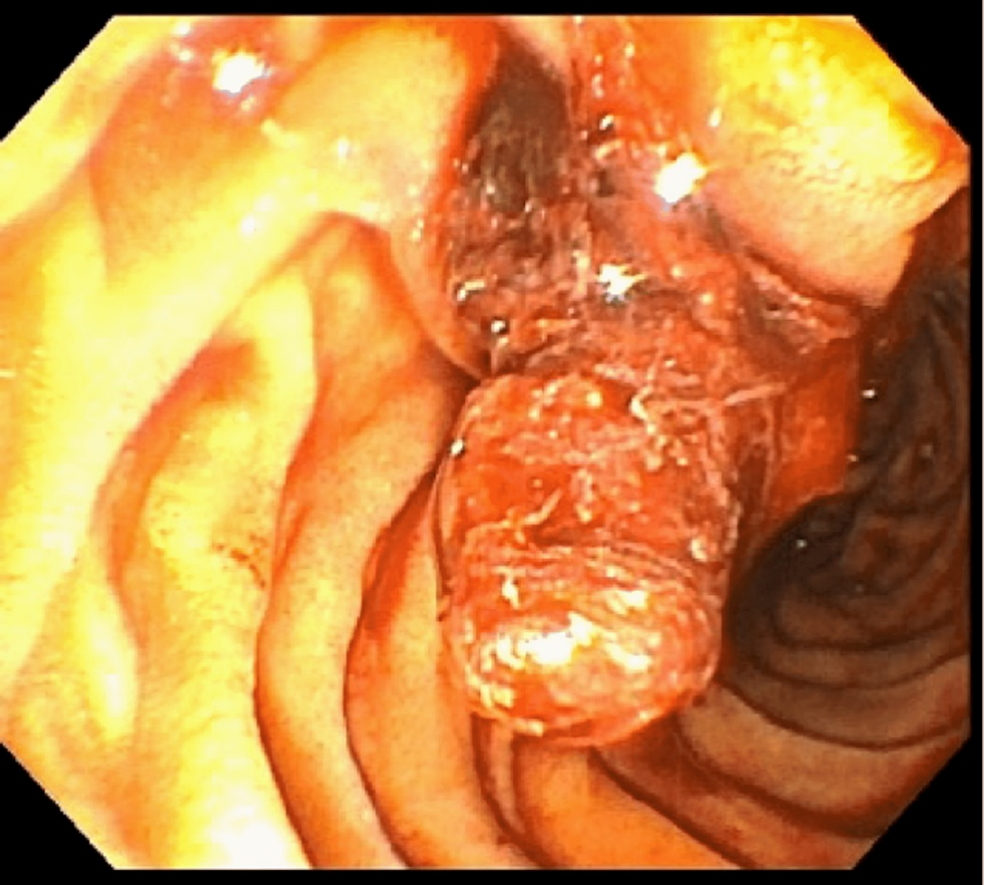
Area of the papilla in the duodenum. The temporary plastic stent is visibly occluded with unclear material.

A magnetic resonance imaging of the abdomen with and without contrast revealed a high-grade CBD stricture within the pancreatic portion and no pancreatic mass. Additionally, it showed restricted diffusion of the pancreas suggestive of immunoglobulin G (IgG) autoimmune pancreatitis. Subsequent endoscopic retrograde cholangiopancreatography (ERCP) and endoscopic ultrasound (EUS) with fine-needle aspiration (FNA) and plastic stent placement were performed. ERCP and EUS findings included obstruction by a possible mass in the lower third of the main bile duct, 3-4 cm above the papilla, which was biopsied, Figure 1. Serum IgG4 was found to be elevated. The biopsy revealed pancreatitis and was negative for malignancy. Due to rising liver transaminases, his stent was exchanged one week later for a metal stent; Figure 2 shows bile duct obstruction. He was later discharged on a three-week high dose steroid taper for IgG4 related autoimmune pancreatitis with a two-week follow-up with surgery. 

**Figure 3:**
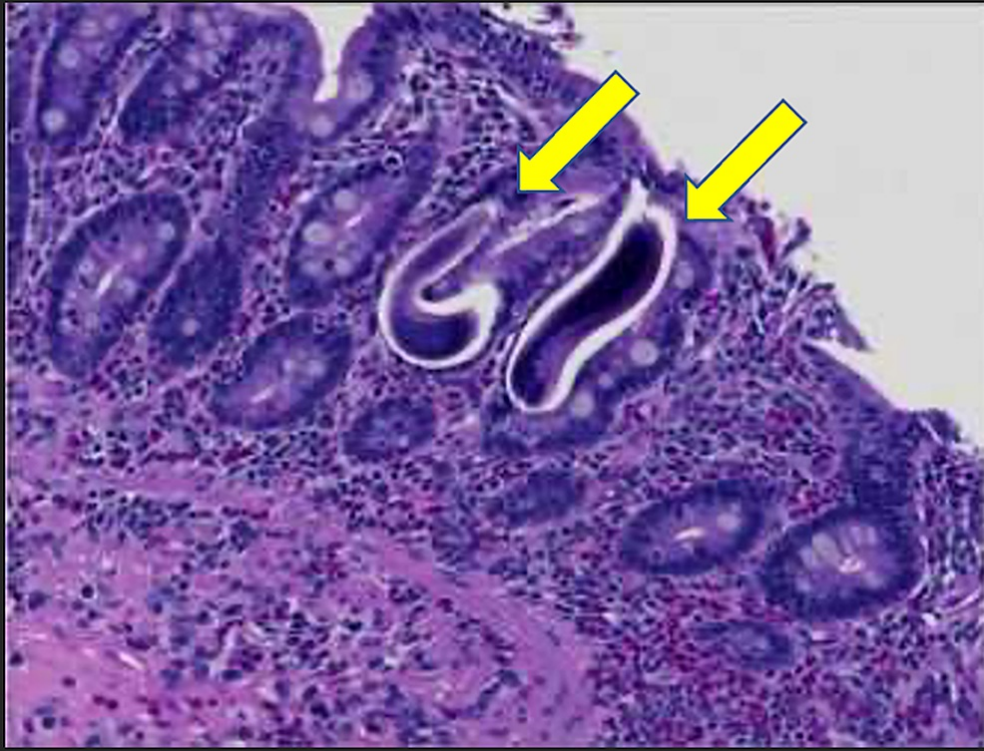
Helminth larva in duodenal wall and ampulla consistent with Strongyloides stercoralis [arrows].

A few weeks later, a Whipple procedure was performed as a more permanent solution to his CBD stricture. The pathological sample reported *S. stercoralis* larvae predominantly involving duodenal mucosa, ampulla, and bile duct lumen, Figure 3. At this point, the Infectious Disease team was consulted. On further chart review, it was recognized that the patient's elevated eosinophil count of 18% was unfortunately overlooked at the time of his initial presentation. The infectious disease team recommended the standard of Strongyloides treatment, oral Ivermectin (200 μg/kg) for two days. The patient tolerated treatment and was later discharged with infectious disease follow-up to confirm clearance of *S. stercoralis*, but he was unfortunately lost to follow-up. 

## Discussion

Our patient was born and raised in an endemic area where he was likely infected early in life and carried this infection for several decades. The patient’s encounter three months before admission, where he was treated with steroids, probably uncovered the parasitic infection. Corticosteroids have been shown to affect the host’s immunity by increasing apoptosis of Th2 cells, therefore reducing the eosinophil count and inhibiting mast cell production [3]. It is speculated that corticosteroids increase ecdysteroid-like substances in the small intestine. These substances act as molting signals and lead to increased production of filariform larva, leading to accelerated autoinfection and dissemination [4].

A confident pathological diagnosis of IgG4-related disease requires the presence of two of the three major histological features; dense lymphoplasmacytic infiltrate, storiform pattern fibrosis, and obliterative phlebitis [5]. A sample attaining the threshold for the IgG4 immunoperoxidase stain does not necessarily qualify for the diagnosis of IgG4-related disease, which is different for every organ [5]. The biopsy in our case did not show any obliterative phlebitis, fibrosis, or lymphoplasmacytic infiltration. However, the immunohistochemical stain for IgG4 showed more than 50 cells/HPF and IgG4/IgG ratio >60%, usually supportive of IgG-related disease. The above findings fall short of the histological features of IgG4-related disease by using the consensus statement for IgG4 disease [5]. Although nonspecific, elevated IgG immunoglobulins are known to occur in *Strongyloides *spp*.* infection [6].

Diagnosis of strongyloidiasis is difficult in uncomplicated infections. Several stool studies are often required to achieve confirmation [7]. Larvae are intermittently shed in a chronically infected immunocompetent patient, and only in hyperinfection syndrome may eggs be present in the stool [2]. New enzyme-linked immunoassay (ELISA) increases diagnostic capabilities, some approaching 100% sensitivity and specificity, but there are still hurdles for serum testing which prevent consistent diagnostic value [1,8]. Many of the available serologic tests are moderately sensitive but cross-react with other helminth parasites, such as schistosomes, and *Ascaris lumbricoides*, decreasing the specificity of the tests [8]. 

*Strongyloides* pancreatitis is an extremely rare complication that has been reported in a handful of cases, around 5-7 in the English and Spanish literature [9-11]. The theorized mechanism for pancreatitis is inflammation of the duodenal ampulla and pancreatic duct and rarely leads to pancreatic duct dilation [9-10]. Like in Machado-Filho et al., our patient had CA 19-9 elevation, and this can be explained due to involvement of the duodenal ampulla and pancreatic duct [11]. Per the available literature, treatment does not seem to be different for this condition [9-11]. In retrospect, the EUS and ERCP could be showing *S. stercoralis *larvae as the cause of obstruction in this case. Given that the stent was discarded, this material was not tested, and we cannot say with 100 percent certainty that it is the *Strongyloides stercoralis* larvae that we see in Figure 2. 

First-line antiparasitic include Ivermectin; a two-day dose has been shown to cure 93% of patients [1]. Albendazole, considered a second-line agent, is used in pregnancy after the first trimester when the fetus is at the least risk of side effects [12]. 

## Conclusions

*Strongyloides stercoralis*, a parasitic nematode, can lead to chronic infection due to its unique lifecycle. Given that chronic infections are usually clinically asymptomatic, uncovering infections after an incidental finding of peripheral blood eosinophilia is not uncommon. Eosinophilia should be a clue for *S. stercoralis*, particularly for patients coming from endemic areas. 
